# High glucose induces Drp1-mediated mitochondrial fission via the Orai1 calcium channel to participate in diabetic cardiomyocyte hypertrophy

**DOI:** 10.1038/s41419-021-03502-4

**Published:** 2021-02-26

**Authors:** Qing-Rui Wu, Dan-Lin Zheng, Pei-Ming Liu, Hui Yang, Lu-An Li, Su-Juan Kuang, Ying-Yu Lai, Fang Rao, Yu-Mei Xue, Ji-Jin Lin, Shuang-Xin Liu, Chun-Bo Chen, Chun-Yu Deng

**Affiliations:** 1 Guangdong Provincial Key Laboratory of Clinical Pharmacology, Research Center of Medical Sciences, Guangdong Provincial People’s Hospital, Guangdong Academy of Medical Sciences, 510080 Guangzhou, Guangdong China; 2 Guangdong Cardiovascular Institute, Guangdong Provincial People’s Hospital, Guangdong Academy of Medical Sciences, 510080 Guangzhou, Guangdong China; 3 School of Medicine, South China University of Technology, 510006 Guangzhou, Guangdong China; 4 School of Biological Science and Engineering, South China University of Technology, 510006 Guangzhou, Guangdong China; 5 School of Pharmaceutical Sciences, Southern Medical University, 510515 Guangzhou, Guangdong China; 6 Department of Nephrology, Guangdong Provincial People’s Hospital, Guangdong Academy of Medical Sciences, 510080 Guangzhou, Guangdong China

**Keywords:** Molecular biology, Cardiac hypertrophy, Pathogenesis

## Abstract

Mitochondrial dysfunction and impaired Ca^2+^ handling are involved in the development of diabetic cardiomyopathy (DCM). Dynamic relative protein 1 (Drp1) regulates mitochondrial fission by changing its level of phosphorylation, and the Orai1 (Ca^2+^ release-activated calcium channel protein 1) calcium channel is important for the increase in Ca^2+^ entry into cardiomyocytes. We aimed to explore the mechanism of Drp1 and Orai1 in cardiomyocyte hypertrophy caused by high glucose (HG). We found that Zucker diabetic fat rats induced by administration of a high-fat diet develop cardiac hypertrophy and impaired cardiac function, accompanied by the activation of mitochondrial dynamics and calcium handling pathway-related proteins. Moreover, HG induces cardiomyocyte hypertrophy, accompanied by abnormal mitochondrial morphology and function, and increased Orai1-mediated Ca^2+^ influx. Mechanistically, the Drp1 inhibitor mitochondrial division inhibitor 1 (Mdivi-1) prevents cardiomyocyte hypertrophy induced by HG by reducing phosphorylation of Drp1 at serine 616 (S616) and increasing phosphorylation at S637. Inhibition of Orai1 with single guide RNA (sgOrai1) or an inhibitor (BTP2) not only suppressed Drp1 activity and calmodulin-binding catalytic subunit A (CnA) and phosphorylated-extracellular signal-regulated kinase (p-ERK1/2) expression but also alleviated mitochondrial dysfunction and cardiomyocyte hypertrophy caused by HG. In addition, the CnA inhibitor cyclosporin A and p-ERK1/2 inhibitor U0126 improved HG-induced cardiomyocyte hypertrophy by promoting and inhibiting phosphorylation of Drp1 at S637 and S616, respectively. In summary, we identified Drp1 as a downstream target of Orai1-mediated Ca^2+^ entry, via activation by p-ERK1/2-mediated phosphorylation at S616 or CnA-mediated dephosphorylation at S637 in DCM. Thus, the Orai1–Drp1 axis is a novel target for treating DCM.

## Introduction

Diabetic cardiomyopathy (DCM), a major cardiovascular complication of diabetes mellitus (DM), is a myocardial dysfunction independent of coronary artery disease and hypertension^1^. DCM is characterized by left ventricular (LV) hypertrophy and cardiac dysfunction. Its pathogenesis is complex, and includes metabolic disorders, mitochondrial dysfunction, impaired cardiomyocyte calcium handling, and inflammation^2^. However, the correlation between calcium handling and mitochondrial dysfunction has not been reported in DCM. In-depth study of the pathogenesis of DCM is helpful to discover new potential therapeutic targets. Therefore, this study aimed to explore the relationship between abnormal calcium regulation and mitochondrial dysfunction of DCM and the potential mechanism.

Mitochondrial homeostasis is essential for maintaining normal physiology in cardiomyocytes with high energy demands, but the underlying mechanism of aberrant mitochondrial morphology in DCM remains unclear. Abnormal mitochondrial morphology indicates an imbalance in mitochondrial dynamics, including mitochondrial fission and fusion. The main proteins that regulate changes in mitochondrial morphology are the dynamic-related GTPases, including dynamic-related protein 1 (Drp1), mitofusin (Mfn), and optic atrophy 1 (Opa1). Drp1 regulates mitochondrial fission, while fusion of the outer and inner mitochondrial membranes is regulated by Mfn1/2 and Opa1, respectively^3^.

Phosphorylation is a post-translational modification that plays an important role in cardiac hypertrophy^4^. Drp1 is mainly regulated by phosphorylation at serine 616 (S616) and S637. For example, mitochondrial fission is driven by extracellular signal-regulated kinase (ERK)-mediated phosphorylation of Drp1 on S616, which plays a role in tumor proliferation^5^. In addition, calcineurin has been reported to induce S637 dephosphorylation, mediating Ca^2+^-induced Drp1-dependent mitochondrial fission^6^. In summary, Drp1-dependent mitochondrial fission is mainly regulated by ERK-mediated phosphorylation at S616 or calcineurin-mediated dephosphorylation at S637. In addition, studies have shown that ERK can be activated by intracellular Ca^2+^ overload^7^. The role of calcineurin as a Ca^2+^/calmodulin-dependent serine/threonine phosphatase in cardiac hypertrophy and remodeling has been extensively investigated^8^. Calcineurin is structurally composed of two subunits^9^: calmodulin-binding catalytic subunit A (CnA) and Ca^2+^-binding regulatory subunit B. This study investigated the role of the calcium-regulated phosphatase CnA and kinase ERK in regulating Drp1 phosphorylation in diabetic cardiomyocytes. We sought to investigate whether Ca^2+^ disorders regulate Drp1 phosphorylation through the activation of ERK or CnA.

Dysregulation of Ca^2+^ handling is observed in DCM. The main mechanism of intracellular calcium signaling is store-operated Ca^2+^ entry (SOCE). The basic unit of SOCE is the accumulation of stromal interaction molecule 1 and Ca^2+^ release-activated calcium channel protein 1 (Orai1) at the endoplasmic reticulum (ER)–plasma membrane junction^10^. Expression of Orai1 protein was first detected in neonate mice heart, but Orai1 expression is more abundant in human myocardial tissue. Besides, the Orai1 channel is important for almost every cell type. In cardiomyocytes, the functions of Orai1 include its mediated SOCE in regulating ER Ca^2+^ content, diastolic Ca^2+^, and cell growth during heart development, but more studies are needed to verify the role of Orai1-mediated Ca^2+^ entry in the adult heart^11^.

Although recent studies have shown that Orai1 expression is upregulated in cardiac hypertrophy associated with MEK/ERK activation^12^, the role of Orai1-mediated SOCE in diabetes-induced cardiomyopathy is unclear. We hypothesized that Orai1-mediated SOCE promotes cardiac hypertrophy induced by high glucose (HG) via regulation of Drp1 phosphorylation. In the present study, we found that Drp1 and Ca^2+^ signaling pathway-related proteins were activated in hypertrophic rat hearts and in the DCM cell model. Moreover, inhibition of Drp1, Orai1, p-ERK1/2, and CnA prevents cardiomyocyte hypertrophy induced by HG by decreasing mitochondrial fission. These data provide evidence that the Orai1–Drp1 pathway may be a target for treating DCM.

## Materials and methods

### Construction of animal models

Ten male Zucker diabetic fat (ZDF) rats and ten male Zucker lean (ZL) rats were obtained from Beijing Vital River Laboratory Animal Technology Co. Ltd (China) at an age of 7 weeks. Six (six-weeks-old) male db/m and db/db mice were purchased from GemPharmatech Co., Ltd (Nanjing, China). The animals were raised alone in specific pathogen-free conditions, with a 12 h light/dark cycle. After 1 week of adaptation, the rats were divided into two groups: ZL, which received a normal diet and ZDF, which received a high-fat diet (Purina 5008). At 7 weeks, six db/db male mice were randomly selected for intraperitoneal injection of Mdivi-1 (10 mg/kg, twice per week, 8 weeks, S716201, Selleck.cn, USA). The animals were considered to have type 2 diabetes at blood glucose concentration ≥11.1 mmol/l. At 18–23 weeks, changes in cardiac function in the two groups of rats were detected by Doppler echocardiography. Briefly, following anesthetization of the rats with 3% isoflurane, chest echocardiography was performed using a Visualsonics Vevo 2100 (Visualsonics Inc., Canada) ultrasound system with a 21-MHz transducer. The recorded parameters were as follows: LV end-diastolic anterior wall thickness, LV wall end diastole, LV ejection fraction, and LV fraction shortening. Then, the body weight (g), heart weight (HW; mg), and HW/tibia length (mg/mm) were measured followed by anesthetization and sacrifice, and LV tissue was excised for analysis. This study was approved by the Ethics Committee and the Teaching and Research Committee (No. GDREC201208A).

### Electron microscopy

The morphology of mouse ventricular mitochondria was observed by electron microscope. In brief, hearts excised from mice were immediately fixed in 2.5% glutaraldehyde (G1102, Servicebio, China). Then, ultrathin sections were examined with a JEM-1400plus transmission electron microscope (Japan Electron Optics Laboratory Co., Ltd, Tokyo). Mitochondrial area and aspect ratio (the ratio of length/width) were quantified by ImageJ. At least 100 randomly selected mitochondria were analyzed for each group.

### Cell culture, infection, and treatment

Neonatal Sprague–Dawley rats (age, 1–3 days) were purchased from The Experimental Animal Center of Southern Medical University (Guangzhou, China). The hearts were removed, cut into pieces, and digested with 0.08% trypsin (T4799, Sigma, USA) and collagenaseII (LS004176, Worthington, USA). The purified cardiomyocytes were obtained by different adherence times, and 10 μM bromodeoxyuridine (S7918, Selleck.cn, USA) was added to inhibit fibroblast proliferation. Neonatal rat cardiomyocytes (NRCMs) were cultured in Dulbecco’s modified Eagle’s medium (DMEM, C11885500BT, Gibco; Thermo Fisher Scientific, USA) supplemented with 10% fetal bovine serum (10099-414, Gibco) and 1% penicillin and streptomycin (15140122, Gibco) at 37 °C and 5% CO_2_. NRCMs were treated with 5.5 mM glucose (normal glucose group), 33 mM mannitol (high mannitol [HM] group), 33 mM glucose (HG group), 10 μM Mdivi-1/BTP2/U0126/cyclosporin A (Mdivi-1/BTP2/U0126/CsA group), or HG and 10 μM Mdivi-1/BTP2/U0126/CsA (HG + Mdivi-1/BTP2/U0126/CsA group). RNA interference was performed with single guide RNA (sgRNA). sgRNAs were introduced by lentiviral vector (genechem.com) according to the manufacturer’s protocol. Mannitol, d-glucose, BTP2, CsA, U0126, and Mdivi-1 were obtained from Sigma.

### Immunofluorescence confocal microscopy

#### Measurement of NRVM size

NRVMs were fixed with 4% formaldehyde for 15 min at 37 °C and washed three times with phosphate-buffered saline. Wheat germ agglutinin (WGA) Texas Red®-X conjugate (W21405, Gibco) was added and the cells were incubated for 10 min at room temperature. After removing the labeling solution and washing cells twice in phosphate-buffered saline, a laser copolymer microscope (SP5-FCS, Leica, Germany) was used to acquire images of labeled cells. NRVM areas were analyzed using the ImageJ software.

#### Mitochondrial morphological measurement

Two hundred nanomoles of Mito-Tracker Red CMXRos (C1049, Beyotime, China) was added and cultures were incubated 10 min at 37 °C according to the instructions. A laser copolymer microscope was used to acquire fluorescence micrographs of labeled mitochondria. MiNA (Mitochondrial Network Analysis), which consists of a set of ImageJ macros, was used to measure mitochondrial morphological parameters from the confocal images^13^.

#### Measurement of mitochondrial fission

Mitochondrial were visualized in NRCMs by staining with 200 nM Mito-Tracker Red CMXRos. Cells with fragmented mitochondria were identified as exhibiting mitochondrial fission.

#### Mitochondrial membrane potential (MMP)

MMP detection reagent JC-1 (C2006, Beyotime) was added and cultures were incubated 15 min at 37 °C according to the manufacturer’s instructions. Red and green fluorescence detected by laser confocal microscopy were from JC-1 aggregates and JC-1 monomers, respectively. ImageJ software was used to conduct semiquantitative analysis of the fluorescence intensity of MMP, and the ratio of red and green fluorescence was measured as the raw data of MMP.

#### Intracellular Ca^2+^ measurement

NRCMs were cultured in DMEM medium with Fluo4/AM (F14201, Invitrogen, USA) at 5 μM for 30 min at 37 °C. NRCMs were washed with Ca^2+^-free Tyrode’s solution (132 mM NaCl, 4.8 mM KCl, 1.2 mM MgCl_2_, 5 mM glucose, 10 mM HEPES, and 1.8 mM CaCl_2_, pH 7.4). Then, 1 μM nifedipine was added to Ca^2+^-free Tyrode’s solution and incubated for 2 min to inhibit L-type Ca^2+^ channel before the addition of thapsigargin (2 μM) to induce ER Ca^2+^ store depletion. At 12 min, Ca^2+^ (CaCl_2_, 2 mM) was added to record the intracellular Ca^2+^ influx mediated by the SOC channel. Fluo4 fluorescence emission was monitored at 525 nm using a confocal laser scanning microscope (SP5-FCS, Leica).

### Total ATP determination

Total ATP was determined using the ATP Assay Kit (S0026, Beyotime, China) according to the manufacturer’s instructions. Briefly, ventricular tissue of mice (~20 mg) was homogenized with 100 μl lysate and centrifuged at 12,000 × *g* at 4 °C for 5 min. The supernatant was mixed with the test solution and the relative light unit was measured by GloMax 20/20 (Promega, USA), and the results were compared to standards. Finally, the concentration of ATP was converted to nmol/mg protein.

### Western blotting

Total protein of ventricular tissues and NRCMs were extracted using RIPA Lysis Buffer (20-188, Millipore, USA) with Protease/Phosphatase Inhibitor Cocktail (5872, CST, USA). The protein concentration of the sample was determined by BCA Protein Assay Kit (P0011, Beyotime), quantified to 20 μg, separated by 10% sodium dodecyl sulfate-polyacrylamide gel electrophoresis, and transferred onto a PVDF membrane (IPVH00010, Millipore). The membrane was sealed with 5% skim milk (232100, BD Biosciences, USA) for 1 h, and probed overnight at 4 °C with primary antibodies against different antigens, including β-myosin heavy chain (β-MHC) (ARP41380, Aviva Systems Biology, USA), atrial natriuretic peptide (ANP) (ab180649, Abcam, USA), Orai1 (ab59330, Abcam), p-ERK1/2 (4370, CST), ERK1/2 (5013, CST), CnA (2614, CST), Drp1 (14647, CST), p-Drp1^S616^ (4494, CST), p-Drp1^S637^ (6319, CST), Mfn2 (9482, CST), and Opa1 (80471, CST). Protein expression levels were normalized to that of glyceraldehyde 3-phosphate dehydrogenase (51332, CST) or α-tubulin (11224-1-AP, Proteintech, USA). Appropriate secondary antibodies were used to bind the primary antibodies for 1 h. The secondary antibodies used were as follows: anti-rabbit IgG-HRP (7074, CST) and anti-mouse IgG-HRP (7076, CST). Bands were visualized by Immobilon Western Chemiluminescent HRP Substrate (WBKLS0500, Millipore) and Exposure machine (ImageQuant LAS500). ImageJ was used to quantify the bands.

### Statistical analysis

SPSS 22.0 statistical software was used for statistical analysis, and GraphPad Prism 5 was used to prepare graphs. All data are expressed as mean ± SEM. Data were initially tested for normality and equal variance by Shapiro–Wilk and equal variance test, respectively. Comparisons between two groups were conducted using an *t* test. To compare the statistical significance of differences among three or more groups, group comparisons were performed using parametric (one-way analysis of variance) and nonparametric (Mann–Whitney *U*) tests when normality and equal variance tests failed. Where variances are homogeneous, the least significant difference test is used, while the Tamhane test is employed when variances cannot be considered homogeneous. For all analysis, statistical significance was reported as follows: ^*^P < 0.05; ^**^P < 0.01; ^***^P < 0.001; n.s., no significance.

## Results

### ZDF rats develop cardiac hypertrophy and exhibit impaired cardiac function

**Fig. 1:**
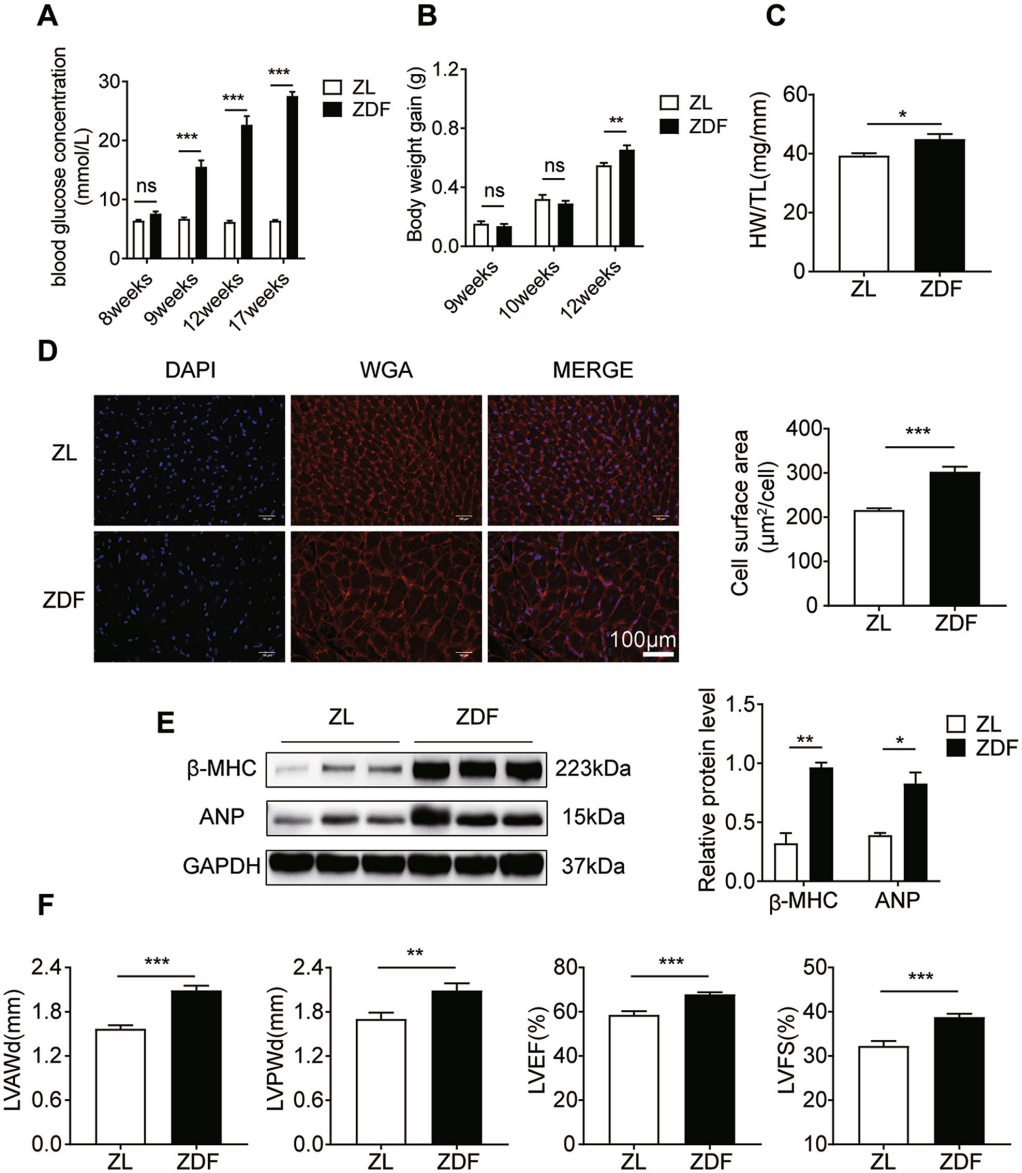
ZDF rats develop cardiac hypertrophy and exhibit impaired cardiac function. **A** Blood glucose at various time points (*n* = 10). **B** Body weight gain (g) = (final body weight − initial body weight)/initial body weight (*n* = 10). **C** Heart weight/tibia length (HW/TL, g/mm) ratio (*n* = 9). **D** WGA staining (red) of the surface membrane of cardiomyocytes, and DAPI staining (blue) of nuclei, scale bars = 100 μm. Quantification of the surface area of cardiomyocytes by WGA staining (*n* = 10). **E** Western blotting images and summarized data showing ANP and β-MHC levels in the ventricular tissue of ZL and ZDF rats (*n* = 6). **F** Echocardiographic parameters of each group of rats (*n* = 10). Data are shown as mean ± SEM. *P < 0.05, **P < 0.01, and ***P < 0.001 vs ZL group.

To investigate cardiac hypertrophy in diabetic rats, we first explored the blood glucose levels and body weights of rats at various time points. Blood glucose was significantly increased in ZDF rats from week 9 compared with ZL rats (Fig. 1A, *P* < 0.001). At the same time, ZDF rats gained significantly more body weight compared with ZL rats at week 12 (Fig. 1B, *P* < 0.01). These results indicated that a diabetic rat model was successfully generated. The HW/tibia length ratio increased significantly in ZDF compared to ZL rats (Fig. 1C, *P* < 0.05). WGA staining showed increased cardiomyocyte size in ZDF rats (Fig. 1D, *P* < 0.001). Further, the protein levels of hypertrophic markers, including ANP and β-MHC, were significantly higher in ZDF rat hearts than that in ZL rat hearts (Fig. 1E, *P* < 0.01). These results imply that ZDF rats develop cardiac hypertrophy. Moreover, echocardiographic assessment of cardiac function showed that LV end-diastolic anterior wall thickness, LV wall end diastole, left ventricular ejection fraction, and left ventricular fractional shortening were increased in ZDF rats compared to ZL rats (Fig. 1F, *P* < 0.01). These results indicated that cardiac hypertrophy and compensatory enhancement of systolic function occurred in ZDF rats.

### Expression levels of mitochondrial dynamics and calcium handling pathway-related proteins in a hypertrophic hearts of ZDF rats

**Fig. 2:**
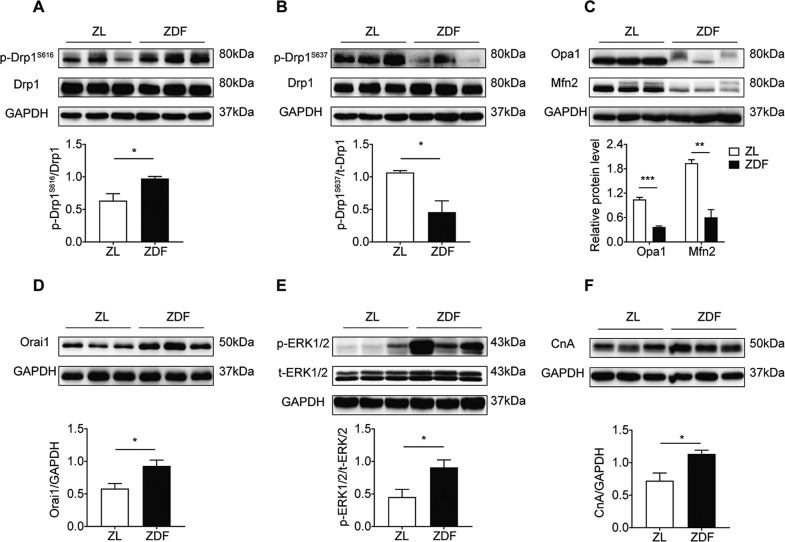
Expression levels of mitochondrial dynamics and calcium handling pathway-related proteins in a hypertrophic hearts of ZDF rats. **A** Western blotting images and summarized data showing phospho-Drp1 (S616) and total Drp1 protein levels in the ventricular tissue of ZL and ZDF rats (*n* = 6). **B** Western blotting images and summarized data showing phospho-Drp1 (S637) and total Drp1 protein levels in the ventricular tissue of ZL and ZDF rats (*n* = 6). **C** Western blotting images and summarized data showing Mfn2 and Opa1 protein levels in the ventricular tissue of ZL and ZDF rats (*n* = 6). **D**–**F** Western blotting images and summarized data showing Orai1, phospho-ERK1/2, total ERK1/2, and CnA protein levels in the ventricular tissue of ZL and ZDF rats (*n* = 6). Data are shown as mean ± SEM. *P < 0.05, **P < 0.01, and ***P < 0.001 vs ZL group.

To investigate the changes in mitochondrial dynamics induced by hyperglycemia in the hearts of ZDF rats, we measured the protein levels of fission and fusion proteins in myocardial tissue. Mitochondrial fission is mainly regulated by cytoplasmic Drp1, and Drp1 is activated by phosphorylation at S616 and dephosphorylation at S637 (ref. ^14^). We found that hyperglycemia increased Drp1 phosphorylation at S616, but reduced phosphorylation at S637 (Fig. 2A, B, *P* < 0.05). The protein expression levels of fusion proteins Opa1 and Mfn2 were significantly decreased in the ZDF group (Fig. 2C, *P* < 0.01). To further investigate the effect of the calcium-related signaling pathway on hyperglycemia-induced cardiac hypertrophy, the protein expression levels of Orai1, CnA, and p-ERK1/2 were examined in myocardial tissue. Interestingly, Orai1, CnA, and p-ERK1/2 protein levels were increased significantly in the ZDF group (Fig. 2D–F, *P* < 0.05). In summary, hyperglycemia increased Orai1, CnA, and p-ERK1/2 protein levels and fission protein Drp1 activity, but decreased fusion protein levels in the hearts of ZDF rats.

### HG induces cardiomyocyte hypertrophy with abnormalities in mitochondrial dynamics and calcium handling

**Fig. 3:**
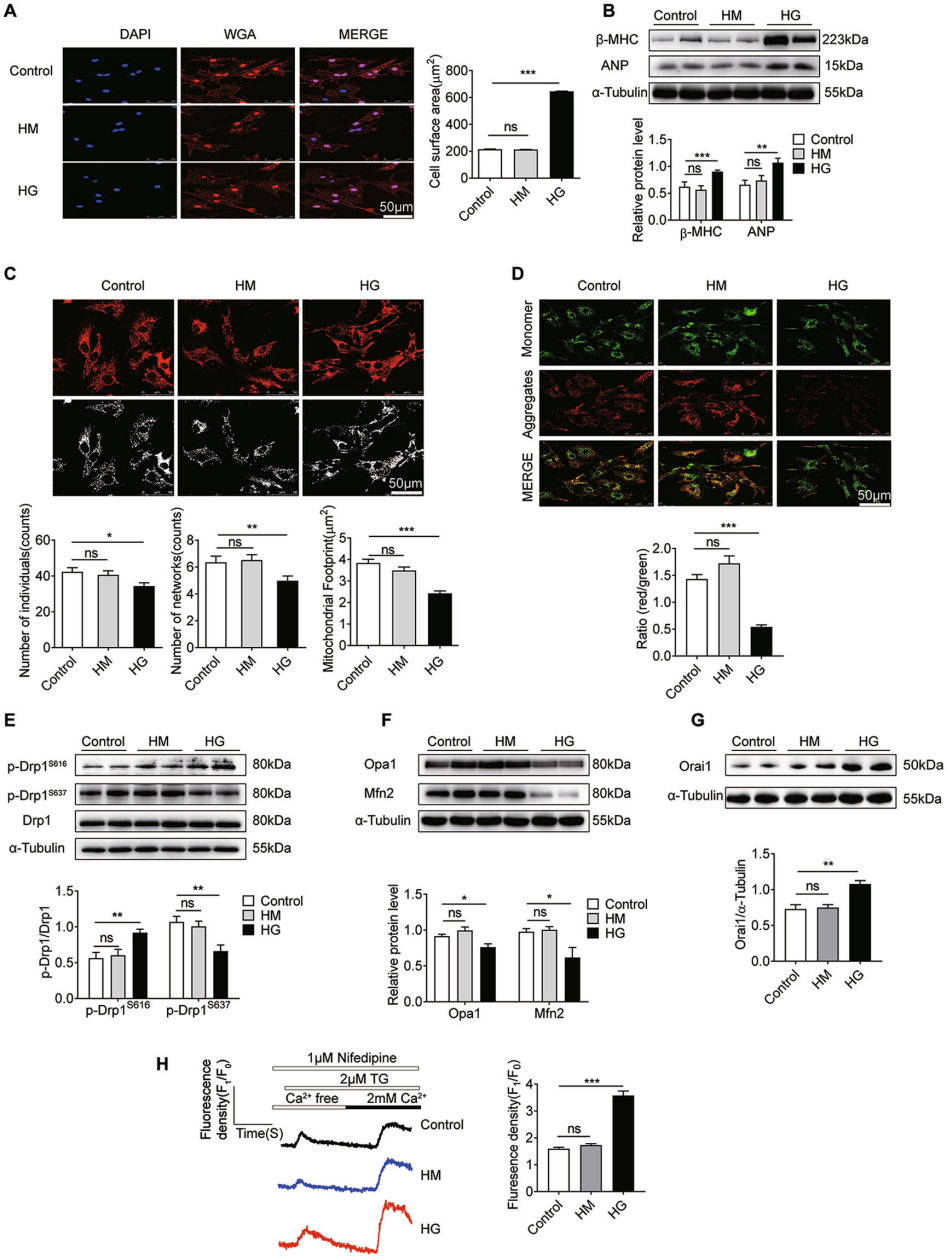
HG induces cardiomyocyte hypertrophy with abnormalities in mitochondrial dynamics and calcium handling. **A** WGA staining (red) of the surface membrane of cardiomyocytes, and DAPI staining (blue) of nuclei. Scale bars = 50 μm. To quantify cardiomyocyte size, HG induced an increase in cardiomyocyte size (*n* = 45 cells). **B** Western blotting images and summarized data showing β-MHC (*n* = 5) and ANP (n = 11) levels in NRCMs. **C** Confocal images of mitochondrial morphology in NRCMs. RGB color, scale bars = 50 μm; 8-bit color, scale bars = 3 μm. There was a main effect of HG on the number of individuals and networks, and the size of the mitochondrial footprint. Overall, HG decreased all mitochondrial network parameters (*n* = 140 cells). **D** JC-1 staining of NRCMs. Red fluorescence is from JC-1 aggregates in healthy mitochondria with polarized inner mitochondrial membranes, while green fluorescence is emitted by cytosolic JC-1 monomers and indicates MMP dissipation. Merged images indicate the co-localization of JC-1 aggregates and monomers. Scale bar = 50 μm. MMP of cardiomyocytes for each group was calculated as the fluorescence ratio of red to green. HG decreased the MMP (*n* = 390 cells). **E** Western blotting images and summarized data showing Drp1 protein level and phosphorylation at S616 (*n* = 6) or S637 (*n* = 8) in NRCMs. **F** Western blotting images and summarized data showing Opa1 (*n* = 4) and Mfn2 (*n* = 3) protein levels in NRCMs. **G** Western blotting images and summarized data showing Orai1 protein levels in NRCMs (*n* = 4). **H** Representative traces of Ca^2+^ influx in NRCMs are shown. Fluorescence intensity measurements of Fluo4-AM revealed the intracellular Ca^2+^ concentration in NRCMs following CaCl_2_ stimulation (*n* = 76 cells). *F*_1_ fluorescence intensity, *F*_0_ baseline fluorescence. Data are shown as mean ± SEM. *P < 0.05, **P < 0.01, and ***P < 0.001; n.s. no significant statistical difference.

To examine whether HG induces cardiomyocyte hypertrophy, we treated primary cultured NRCMs with 33 mM glucose (HG) for 72 h. As shown in Fig. 3A, the NRCM area detected by WGA staining was significantly increased in the HG group (*P* < 0.001). Furthermore, protein expression of the cardiac hypertrophic markers β-MHC and ANP in NRCMs increased significantly in the HG group (Fig. 3B, *P* < 0.01), indicating that HG induced NRCM hypertrophy.

To study mitochondrial functional and morphological changes in NRCMs caused by HG, we determined mitochondrial membrane potential (MMP) and mitochondrial network parameters in NRCMs. In the HG group, NRCM mitochondria tended to be fragmented (decreased number of individuals, networks, and mitochondrial footprint) and showed decreased MMP (Fig. 3C, D, *P* < 0.01). Furthermore, we found that HG increased the phosphorylation of Drp1 at S616, but decreased phosphorylation at S637 (Fig. 3E, *P* < 0.01). Protein expression levels of the fusion protein Opa1 and Mfn2 were significantly decreased in the HG group (Fig. 3F, *P* < 0.05). In summary, HG increased Drp1-dependent mitochondrial fission and decreased mitochondrial fusion, resulting in mitochondrial dysfunction and morphological fragmentation.

To further identify the effect of HG on Orai1 expression and channel function, we also determined the protein expression of Orai1 in NRCMs. We found that the expression of Orai1 was significantly greater in the HG group compared with the control group (Fig. 3G, *P* < 0.01). We also determined Orai1-mediated Ca^2+^ entry using the Fluo4/AM dye in NRCMs. As shown in Fig. 3H, the L-type Ca^2+^ channel was blocked with 1 μM nifedipine. Subsequent addition of 2 mM calcium 10 min after the addition of 2 μM thapsigargin resulted in a larger and sustained increase in Ca^2+^ entry through the Orai1 channel. Stronger Ca^2+^ fluorescence was observed in the HG group compared to that in the control groups following stimulation with CaCl_2_ (*P* < 0.001). There was no statistical difference between the control and HM groups (*P* > 0.05). Accordingly, we confirmed that HG can induce Orai1-mediated intracellular Ca^2+^ overload by upregulating Orai1.

### Mdivi-1 alleviates cardiomyocyte hypertrophy induced by HG by decreasing Drp1-dependent mitochondrial fission

**Fig. 4:**
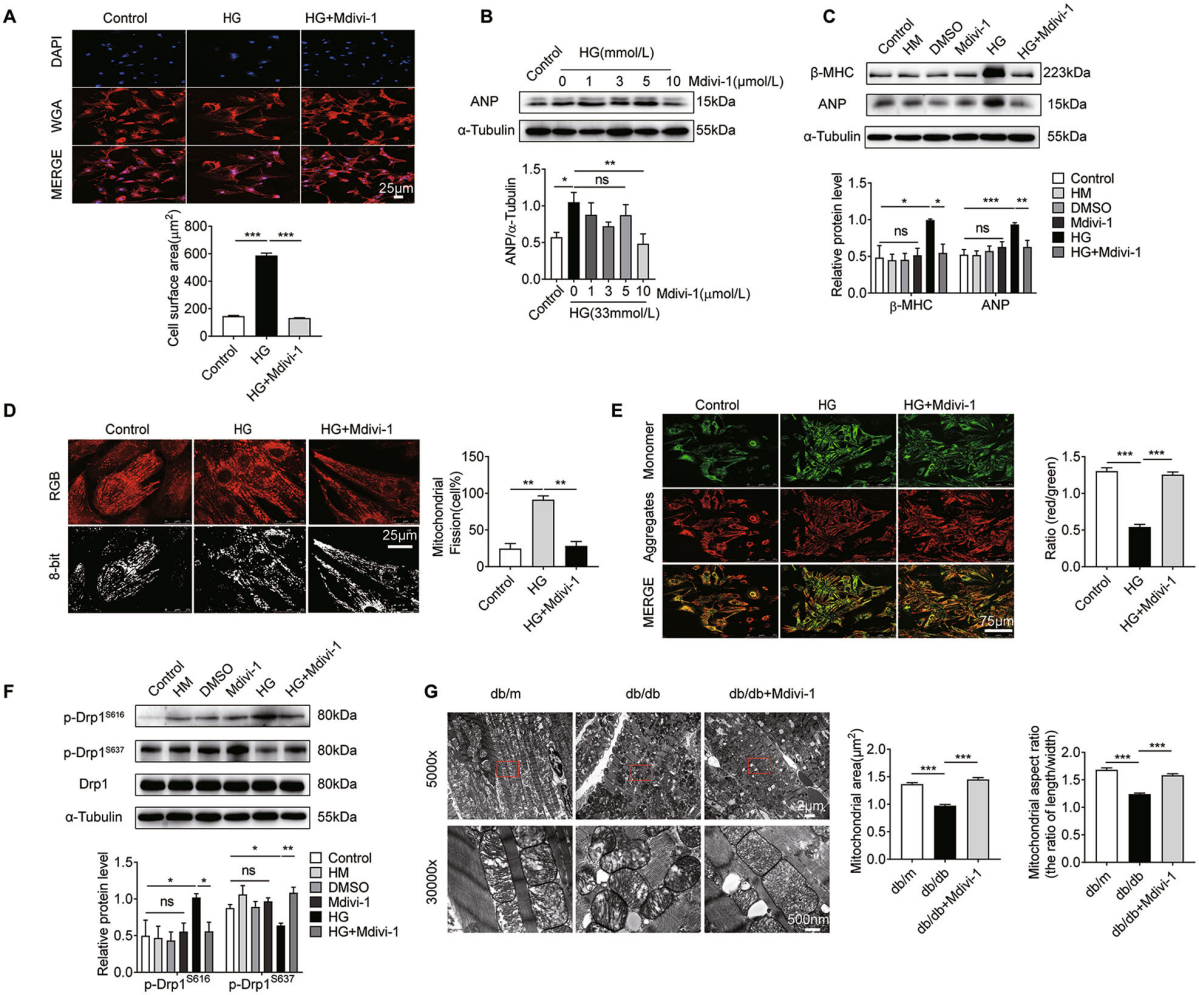
Mdivi-1 alleviates cardiomyocyte hypertrophy induced by HG by decreasing Drp1-dependent mitochondrial fission. **A** WGA staining (red) of the surface membrane of cardiomyocytes, and DAPI staining (blue) of nuclei. Scale bars = 25 μm. To quantify cardiomyocyte size, HG induced an increase in cardiomyocyte size (*n* = 104 cells). **B** Changes in ANP expression in NRCMs treated with different concentrations of Mdivi-1 (*n* = 4). **C** Changes in β-MHC (*n* = 4) and ANP (*n* = 6) levels in NRCMs treated with 10 μM Mdivi-1. **D** Mitochondrial fission was detected by Mito-Tracker Red staining. Mitochondrial fission was quantified by counting the proportion of cells with fragmented mitochondria (*n* = 138 cells). Scale bar = 25 μm. **E** MMP of NRCMs was detected by JC-1 staining (10 μg/ml) using confocal microscopy. Scale bar = 75 μm. MMP of cardiomyocytes for each group was calculated as the fluorescence ratio of red to green. HG decreased the MMP (*n* = 290 cells). **F** Western blotting images and summarized data showing phospho-Drp1 (S616/S637) and total Drp1 protein levels in NRCMs (*n* = 3). **G** Representative transmission electron microscopy images of mitochondria and sarcomere ultrastructure. Scale bars = 2 μm (×5000) or 500 nm (×30,000). Mean area of mitochondria and mitochondrial aspect ratio. At least 100 randomly selected mitochondria were analyzed for each group. Data are shown as mean ± SEM. *P < 0.05, **P < 0.01, and ***P < 0.001; n.s. no significant statistical difference.

The Mdivi-1 is a selective inhibitor of Drp1, and has been reported to affect ischemia/reperfusion injury and cardiac hypertrophy in animal models^15^. We treated NRCMs with Mdivi-1 to determine whether it could block cardiomyocyte hypertrophy induced by HG. To this end, the cell surface area and the protein expression levels of β-MHC and ANP were assessed in NRCMs. As shown in Fig. 4A, Mdivi-1 significantly inhibited the increased cardiomyocyte area induced by HG (*P* < 0.001). Meanwhile, the upregulation of ANP in NRCMs induced by HG was significantly inhibited by 10 μM Mdivi-1 (Fig. 4B, *P* < 0.01). Furthermore, Mdivi-1 significantly reduced the upregulation of β-MHC and ANP induced by HG (Fig. 4C, *P* < 0.01). These results suggest that Mdivi-1 could alleviate HG-induced cardiac hypertrophy.

Next, we explored the cellular mechanisms by which Mdivi-1 protects against HG-induced cardiomyocyte hypertrophy. As shown in Fig. 4D, mitochondrial fission in Mdivi-1-treated cells was decreased compared with the HG group (*P* < 0.01). Furthermore, the decrease in MMP in HG conditions was dramatically inhibited by Mdivi-1 compared with the HG group (Fig. 4E, *P* < 0.001). These results indicated that Mdivi-1 improved HG-induced mitochondrial dysfunction.

We then explored the expression of phospho-Drp1 (S616 and S637) and total Drp1 protein levels in NRCMs. As shown in Fig. 4F, Mdivi-1 significantly inhibited the upregulation of p-Drp1^S616^ and downregulation of p-Drp1^S637^ induced by HG (*P* < 0.01). Total Drp1 showed no significant changes. No obvious difference was observed between the control, HM, dimethyl sulfoxide, and Mdivi-1 groups (*P* > 0.05). Collectively, these results indicated that Mdivi-1 inhibited Drp1 phosphorylation at S616 and increased Drp1 phosphorylation at S637, preventing mitochondrial fission induced by HG.

To further test this in vivo, we explored the therapeutic role of Mdivi-1 on DCM. We first examined the effect of Mdivi-1 injection on cardiac hypertrophy in diabetic mice. We then investigated the effect of Mdivi-1 on mitochondrial morphology and ATP level in db/db diabetic mice. As shown in Fig. S1A, protein levels of β-MHC and ANP were increased in db/db mice hearts compared with db/m mice, but decreased in db/db mice after Mdivi-1 treatment. Consistent with in vitro results, p-Drp1^S616^ expression was increased and p-Drp1^S637^ expression was decreased in diabetic mice, and Mdivi-1 intervention in vivo reversed these changes (Fig. S1B). In addition, direct ATP measurement revealed that the myocardial ATP concentration was significantly reduced in db/db mice, and a significant increase in db/db + Mdivi-1 mice was observed (Fig. S1C, *P* < 0.05).

The electron microscopy showed that the area and aspect ratio of mitochondria were significantly decreased in db/db compared with db/m group, which indicated that mitochondrial fission was broadly raised. This alteration was improved by Mdivi-1 injection (Fig. 4G, *P* < 0.001). No obvious difference was observed between the db/m and db/db + Mdivi-1 groups (*P* > 0.05).

### Orai1 inhibition prevents HG-induced cardiomyocyte hypertrophy and mitochondrial dysfunction in NRCMs

**Fig. 5:**
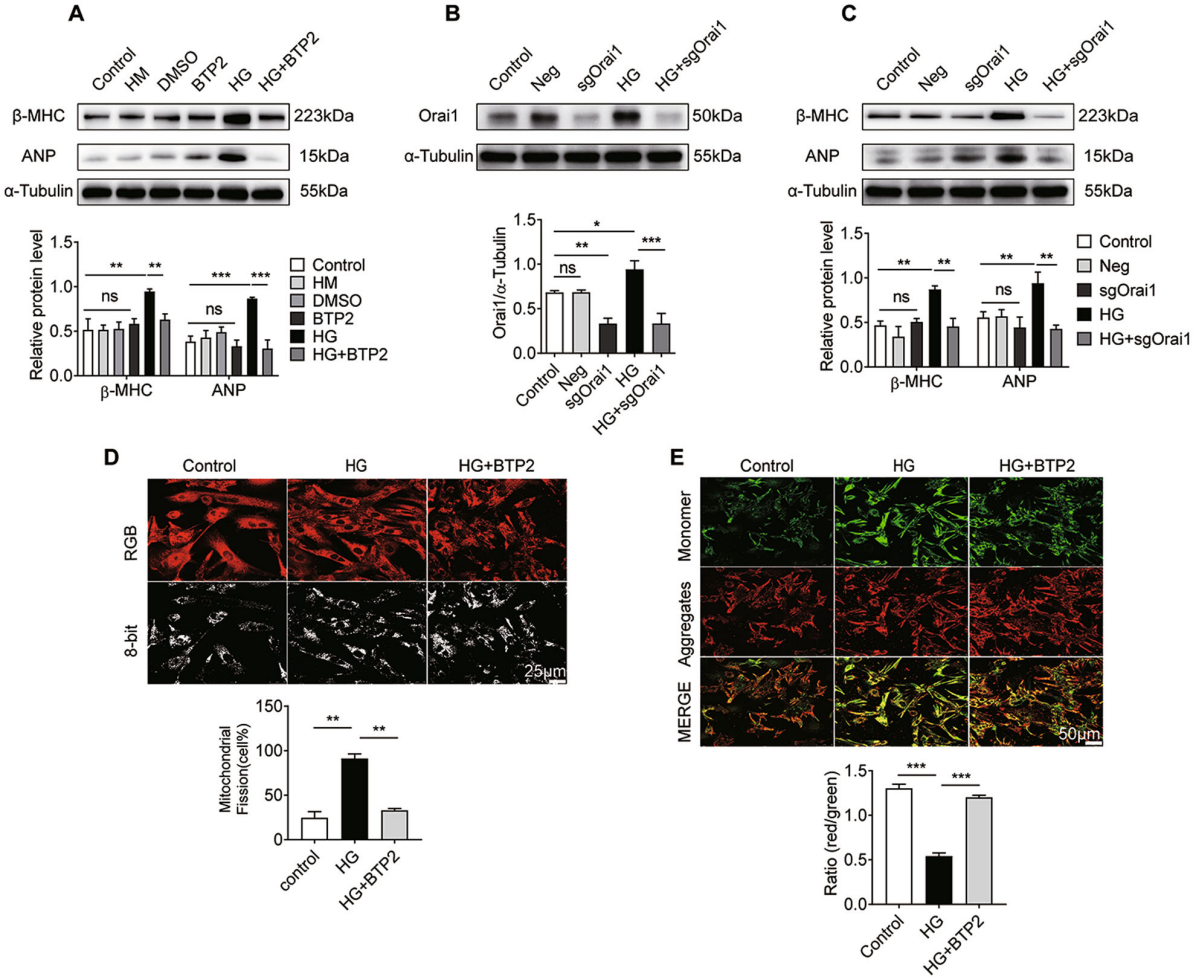
Orai1 inhibition prevents HG-induced cardiomyocyte hypertrophy and mitochondrial dysfunction in NRCMs. **A** Changes of β-MHC (*n* = 4) and ANP (*n* = 5) levels in NRCMs treated with BTP2 at 10 μM. **B**, **C** Changes in Orai1 (*n* = 4), β-MHC (*n* = 4), and ANP (*n* = 5) levels in NRCMs infected with sgOrai1 at 80 MOI for 72 h. **D** Mitochondrial fission was detected by Mito-Tracker Red staining. Scale bar = 25 μm. Mitochondrial fission was quantified by counting the proportion of cells with fragmented mitochondria (*n* = 137 cells). **E** MMP of NRCMs was detected by JC-1 staining (10 μg/ml) using confocal microscopy. Scale bar = 50 μm. MMP of cardiomyocytes for each group was calculated as the fluorescence ratio of red to green. HG decreased the MMP (*n* = 180 cells). Data are shown as mean ± SEM. *P < 0.05, **P < 0.01, and ***P < 0.001; n.s. no significant statistical difference.

We previously showed that HG induced Orai1-mediated intracellular Ca^2+^ overload by upregulating Orai1 expression. Next, we investigated whether inhibition of Orai1 prevents HG-induced cardiomyocyte hypertrophy in NRCMs. We used BTP2 to block the Orai1 channel, which is a Ca^2+^ release-activated Ca^2+^ channel inhibitor. We found that BTP2 significantly reduced the upregulation of β-MHC and ANP induced by HG (Fig. 5A, *P* < 0.01). These results suggest that BTP2 could alleviate HG-induced cardiac hypertrophy. Further, the Cas9/sgRNA technique was used to knock down Orai1 in cardiomyocytes. As shown in Fig. 5B, the protein expression levels of Orai1 in the sgOrai1 group were significantly decreased compared with the Neg group (*P* < 0.01). Meanwhile, compared to the HG group, Orai1 protein expression was obviously decreased in the HG + sgOrai1 group (*P* < 0.001). Furthermore, Orai1 knockdown inhibited HG-induced β-MHC and ANP expression (Fig. 5C, *P* < 0.01). According to these results, we concluded that high expression of Orai1 might be a promoter in cardiomyocyte hypertrophy induced by HG.

We also studied the effects of Orai1 inhibition on mitochondrial dysfunction induced by HG in NRCMs. As shown in Fig. 5D, much shorter and smaller mitochondria were prevalent in the HG group compared with the control group, indicating mitochondrial fragmentation. Quantitative results showed that mitochondrial fission in BTP2-treated cells was decreased compared with the HG group (*P* < 0.01). Furthermore, the decrease in MMP in the HG condition was dramatically inhibited by BTP2 compared with the HG group (Fig. 5E, *P* < 0.001). These results indicated that BTP2 improved HG-induced mitochondrial dysfunction.

### Orai1 might contribute to HG-induced cardiomyocyte hypertrophy via activation of CnA, ERK-Drp1 pathways

**Fig. 6:**
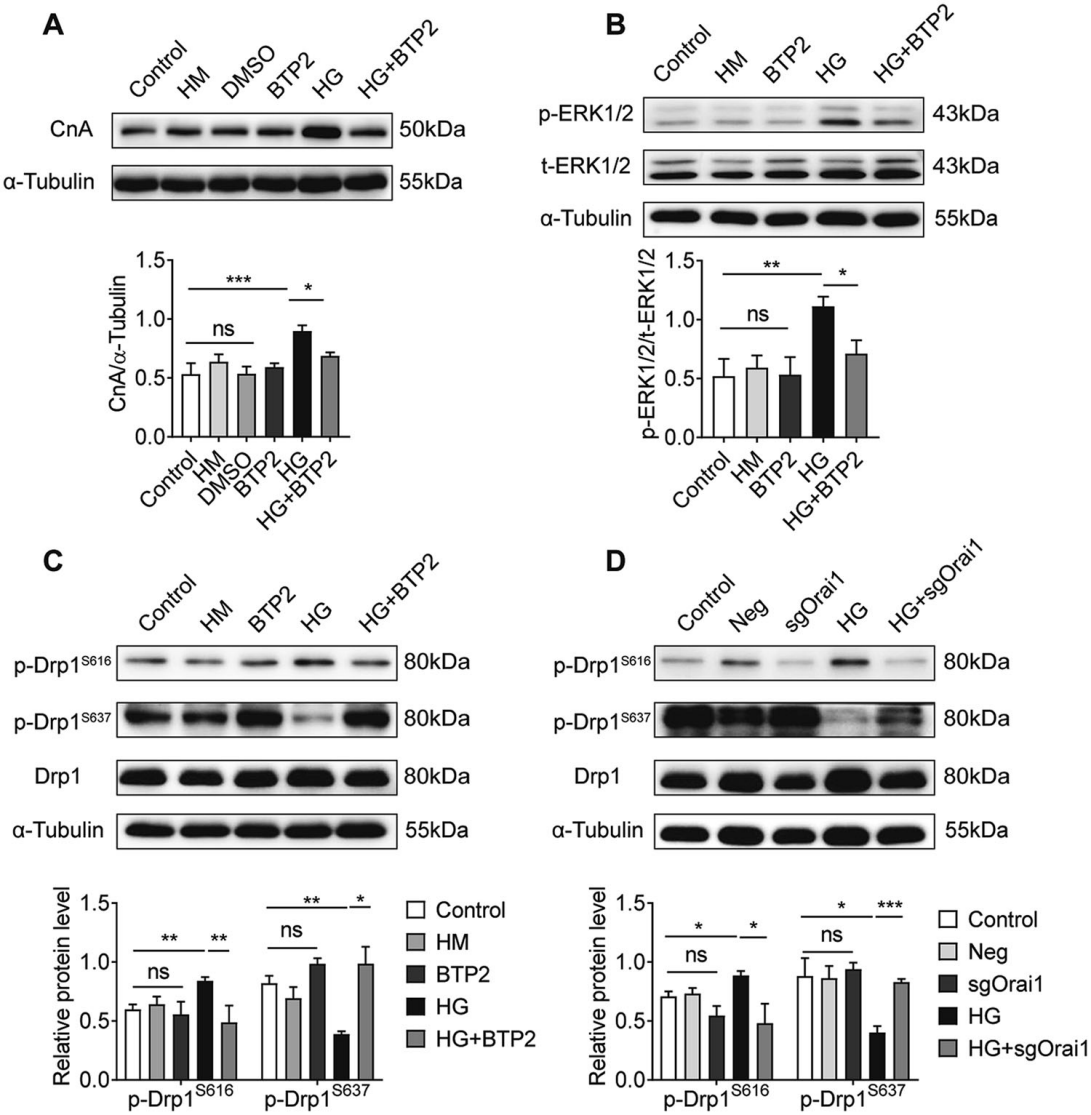
Orai1 might contribute to HG-induced cardiomyocyte hypertrophy via activation of CnA, ERK-Drp1 pathways. **A** Changes in CnA levels in NRCMs treated with 10 μM BTP2 (*n* = 4). **B** Changes of p-ERK1/2 and total ERK1/2 protein levels in NRCMs treated with BTP2 10 μM (*n* = 3). **C** Western blotting images and summarized data showing p-Drp1^S616^ (*n* = 4) and p-Drp1^S637^ (*n* = 3) protein levels in NRCMs treated with 10 μM BTP2. **D** Changes of p-Drp1^S616^ (*n* = 4) and p-Drp1^S637^ (*n* = 4) levels in NRCMs infected with sgOrai1 at 80 MOI for 72 h. The protein levels of phospho-Drp1 (S616/S637) were normalized by total Drp1. Data are shown as mean ± SEM. *P < 0.05, **P < 0.01, and ***P < 0.001; n.s: no significant statistical difference.

To understand the cellular mechanisms by which Orai1-mediated Ca^2+^ entry contributes to HG-induced cardiomyocyte hypertrophy, we aimed to determine whether CnA and ERK-Drp1 pathways were involved in this process. Therefore, western blotting was used to study the effects of Orai1 inhibitor BTP2 (10 µM) treatment on CnA and the phosphorylation of ERK and Drp1 in NRCMs. We found that BTP2 significantly reduced the upregulation of CnA in hypertrophic cells induced by HG (Fig. 6A, *P* < 0.05). BTP2 also significantly inhibited the upregulation of p-ERK1/2 induced by HG (*P* < 0.05). Total ERK1/2 showed no significant changes (Fig. 6B, *P* > 0.05). Furthermore, BTP2 significantly inhibited the upregulation of p-Drp1^S616^ and downregulation of p-Drp1^S637^ induced by HG (Fig. 6C, *P* < 0.01). Orai1 knockdown also significantly inhibited the upregulation of p-Drp1^S616^ and downregulation of p-Drp1^S637^ induced by HG (Fig. 6D, *P* < 0.01). Total Drp1 showed no significant changes (*P* > 0.05). No obvious difference was observed between the control, Neg, and sgOrai1 groups (*P* > 0.05). These data demonstrated that CnA, ERK1/2, and Drp1 may be downstream pathways of Orai1-mediated Ca^2+^ influx; however, the role of CnA and ERK1/2 in regulating Drp1 phosphorylation in HG-induced cardiomyocyte hypertrophy remains unclear.

### CnA and p-ERK1/2 contribute to HG-induced cardiomyocyte hypertrophy by regulating Drp1 phosphorylation

**Fig. 7:**
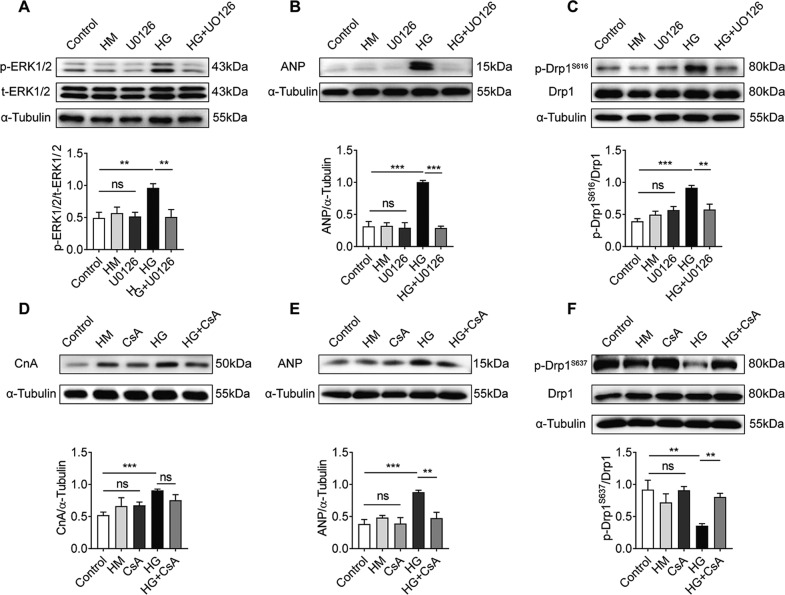
CnA and p-ERK1/2 contribute to HG-induced cardiomyocyte hypertrophy by regulating Drp1 phosphorylation. **A** Changes in p-ERK1/2 levels in NRCMs treated with U0126 at 10 μΜ. Protein levels of p-ERK1/2 were normalized by total ERK1/2 (*n* = 4). **B** Changes in ANP levels in NRCMs treated with U0126 at 10 μM (*n* = 7). **C** Changes in p-Drp1^S616^ levels in NRCMs treated with 10 μΜ U0126. The protein level of p-Drp1^S616^ was normalized by total ERK1/2 (*n* = 6). **D** Changes in CnA levels in NRCMs treated with 10 μΜ CsA (*n* = 3). **E** Changes of ANP level in NRCMs treated with CsA at 10 μM (*n* = 5). **F** Changes in p-Drp1^S637^ levels in NRCMs treated with 10 μΜ CsA. Quantification of Drp1 phosphorylation at S637 corrected for total Drp1 (*n* = 4). Data are shown as mean ± SEM. *P < 0.05, **P < 0.01, and ***P < 0.001; n.s. no significant statistical difference.

To clarify the role of CnA and p-ERK1/2 in regulating Drp1 phosphorylation in HG-induced cardiomyocyte hypertrophy, we treated NRCMs with the CnA inhibitor CsA or the p-ERK1/2 inhibitor U0126, respectively. As shown in Fig. 7A, following the addition of 10 μM U0126, p-ERK1/2 levels in NRCMs were significantly reduced compared with HG-treated samples (*P* < 0.01). Total ERK1/2 showed no significant changes (*P* > 0.05). We found that U0126 could significantly inhibit the upregulation of ANP induced by HG (Fig. 7B, *P* < 0.001). Meanwhile, UO126 significantly inhibited the phosphorylation of Drp1 at S616 in a HG environment (Fig. 7C, *P* < 0.01). Notably, HG increased the abundance of CnA, but the expression of CnA was not influenced by CsA treatment (Fig. 7D, *P* < 0.01). Similarly, the expression of ANP in NRCMs treated with CsA was significantly reduced compared with the HG group (Fig. 7E, *P* < 0.01). CsA significantly induced phosphorylation of Drp1 at S637 in a HG environment (Fig. 7F, *P* < 0.01). Overall, these data show that mitochondrial fission induced by phosphorylation of Drp1 at S616 or dephosphorylation at S637 is a critical event in HG-induced cardiomyocyte hypertrophy by p-ERK1/2 or CnA activation.

## Discussion

**Fig. 8:**
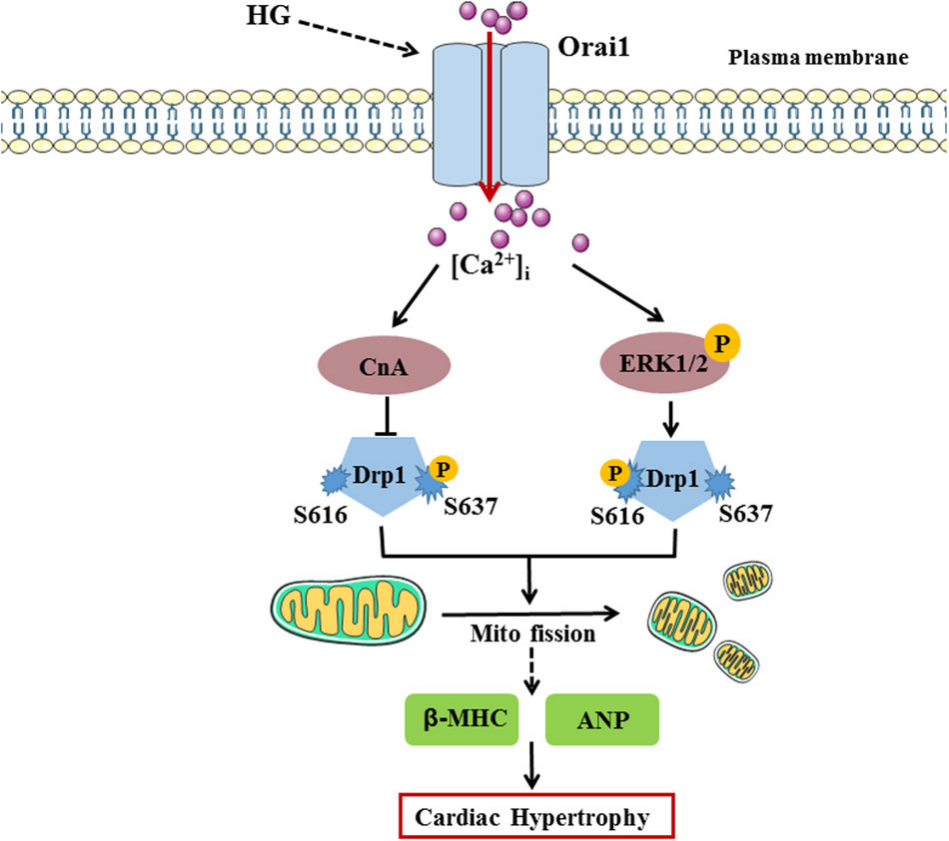
Schematic diagram depicting proposed Orai1–Drp1 signaling pathway in cardiomyocyte hypertrophy induced by HG. HG stimulation induces upregulation of Orai1. Then, Orai1-mediated Ca^2+^ entry targets CnA to inhibit Drp1 phosphorylation at S637, inducing cardiac hypertrophy. Orai1-mediated Ca^2+^ entry also targets p-ERK1/2 to induce Drp1 phosphorylation at S616. Phosphorylation of Drp1 at S616 is increased, while phosphorylation of Drp1 at S637 is decreased, promoting mitochondrial fission and accelerating HG-induced cardiac hypertrophy. Inhibition of the Orai1–Drp1 signaling pathway could prevent cardiac hypertrophy induced by HG.

Advanced DCM characterized by LV hypertrophy and hyperglycemia is one of the main causes of metabolic changes in diabetes^1^. Therefore, we cultured NRCMs in HG medium to establish a cell model for studying the pathogenesis of DCM. We report a novel mechanism that contributes to HG-induced cardiac hypertrophy (Fig. 8). We found that HG increased Orai1, CnA, and p-ERK1/2 protein levels and Drp1 activity in vivo and in vitro. Importantly, we showed for the first time that inhibition of Orai1-mediated Ca^2+^ entry reduces HG-induced cardiomyocyte hypertrophy by decreasing Drp1-dependent mitochondrial fission, and that Drp1 activity is regulated by CnA and p-ERK1/2. This study provides new insights into the role and mechanism of Orai1 in DCM via mitochondrial dysfunction.

ZDF rats are generally considered a well-characterized model of type 2 diabetes with significant cardiovascular dysfunction. In the present study, as expected, the cardiac function of ZDF rats was impaired. The ratio of HW/TL and the expression level of hypertrophy-related proteins in ZDF rats were significantly increased, indicating that the cardiac dysfunction in diabetic rats primarily manifested as cardiac hypertrophy. Notably, we found that Drp1 and Orai1 signaling pathway-related proteins were activated in hypertrophic rat hearts. However, the detailed mechanism of the Drp1 and Orai1 signaling pathway in DCM remains obscure.

The role of mitochondrial dynamics-related proteins in cardiomyopathy has been studied^16^. The main mitochondrial fusion protein Mfn2 was downregulated in cardiac hypertrophy. Opa1 protein was significantly decreased after heart failure and myocardial infarction. Drp1 contributes to the promotion of hypertensive cardiac hypertrophy^17^. Drp1 activity is activated by phosphorylation at S616 and dephosphorylation at S637 (ref. ^18^). Moreover, previous reports have shown that inhibition of Drp1-mediated mitochondrial fission could reduce mitochondrial dysfunction and cardiac dysfunction in diabetic mice^19^. Notably, the effect of phosphorylation at different sites of Drp1 on DCM is unknown. In the present study, we showed that HG induces cardiomyocyte hypertrophy with abnormalities in mitochondrial dynamics. HG promoted Drp1-dependent mitochondrial fission and decreased mitochondrial fusion, leading to mitochondrial dysfunction. Thus, we considered that inhibition of Drp1 could attenuate the cardiomyocyte hypertrophy induced by HG. Mdivi-1 is a cell-permeable selective inhibitor of Drp1-mediated mitochondrial fission. A previous study indicated that Mdivi-1 improves mitochondrial function and cardiac function after myocardial infarction in diabetic mice^20^. However, the mechanisms through which this compound functions directly in DCM remain unknown. Importantly, we found that Mdivi-1 could inhibit HG-induced cardiomyocyte hypertrophy by decreasing Drp1 phosphorylation at S616 or increasing phosphorylation at S637 in NRCMs. In addition, treatment with Mdivi-1 restores mitochondrial morphology and ATP production along with the inhibition of cardiac hypertrophy in the diabetic mice. It turns out that Drp1-mediated mitochondrial fission promotes diabetic cardiac hypertrophy by decreasing myocardial energy storage. However, the underlying mechanism by which HG regulates Drp1 phosphorylation remains unclear.

A previous study showed that disorders of mitochondrial dynamics are related to abnormal calcium levels^21^. We have shown that HG induced expression of Orai1 and Orai1-mediated Ca^2+^ entry. But it is unclear whether Orai1-mediated Ca^2+^ entry is associated with mitochondrial dysfunction in DCM. Previous studies showed that inhibition of the upregulation of stromal interaction molecule 1/Orai1 prevents cardiac hypertrophy^22^ and that SOCE plays an important role in activating transcriptional pathways associated with cardiomyocyte hypertrophy^23^. Our study demonstrated that inhibition of Orai1-mediated SOCE by BTP2 prevents cardiomyocyte hypertrophy and mitochondrial dysfunction induced by HG. In addition, knockdown of Orai1 attenuated HG-induced cardiomyocyte hypertrophy. Together, these studies indicate the involvement of Orai1-mediated Ca^2+^ entry in HG-induced cardiomyocyte hypertrophy and mitochondrial dysfunction.

Furthermore, we found that BTP2 significantly inhibited the upregulation of CnA and p-ERK1/2 in NRCMs treated with HG. Consistent with previous results, Orai1 knockdown may play a role in myocardial hypertrophy by reducing CnA and ERK1/2 activity^24–26^. Restoring intracellular Ca^2+^, downregulating the expression of CnA, inhibiting the phosphorylation of Drp1 at S616, and increasing the phosphorylation of Drp1 at S637 could prevent mitochondrial fission in cardiomyocytes^27^. Consistent with previous results, this study showed that both BTP2 treatment and Orai1 knockdown significantly inhibited the increase in p-Drp1^S616^ expression and decrease in p-Drp1^S637^ expression induced by HG. Next, we verified that Orai1 may be involved in the regulation of Drp1 activity through CnA or ERK pathways in DCM.

Previous studies have shown that inhibiting the levels of Ca^2+^ in the cytoplasm contributes to reduce mitochondrial fission and improve mitochondrial and heart function^28^. In addition, ERK1/2 promotes Drp1 phosphorylation, leading to mitochondrial dysfunction in heart failure^29^. Studies have been reported that the ERK signaling pathway induces phosphorylation of Drp1 at S616 (ref. ^30^). Activation of ERK-mediated mitochondrial Drp1 phosphorylation at S616 promotes chemotherapy resistance in colorectal cancer^31^ and inhibition of p-ERK1/2 by U0126 suppresses mitochondrial fission by decreasing Drp1 phosphorylation at S616 in Huntington’s disease mutant cells^32^. Consistent with previous results, we found that Drp1 phosphorylation at S616 is increased in hypertrophic cardiomyocytes induced by HG; this effect was abolished by treatment with U0126. In addition, previous studies have shown that the Ca^2+^-dependent phosphatase calcineurin regulates translocation of Drp1 to mitochondria through dephosphorylation of S637 in HeLa cells^6^. Most functional studies of calcineurin have been performed with its inhibitor CsA (ref. ^33^). This study found that CsA could significantly inhibit HG-induced cardiomyocyte hypertrophy by increasing mitochondrial fission via promotion of dephosphorylation of Drp1 at S637.

In summary, we believe that HG could induce intracellular Ca^2+^ overload by upregulating Orai1. Orai1-mediated Ca^2+^ influx activates ERK or CnA-Drp1 pathway, leading to diabetic cardiac hypertrophy via promoting mitochondrial fission. Inhibition of Orai1-Ca^2+^-CnA or ERK-Drp1 signaling pathways could prevent cardiomyocyte hypertrophy induced by HG. Our results suggest that targeting Orai1–Drp1 axis may offer a promising approach to ameliorate the cardiac hypertrophy associated with type 2 DM.

## Supplementary information

Supplementary figure and figure legend
