# Supplementary material for: High glucose induces Drp1-mediated mitochondrial fission via the Orai1 calcium channel to participate in diabetic cardiomyocyte hypertrophy
Source: Cell Death Dis. 2021 Feb 26;12(2):216. doi: 10.1038/s41419-021-03502-4 (PMC7910592; doi:10.1038/s41419-021-03502-4)
Supplement: Supplementary file 1 — Supplementary figure and figure legend [file 41419_2021_3502_MOESM1_ESM.docx]

**
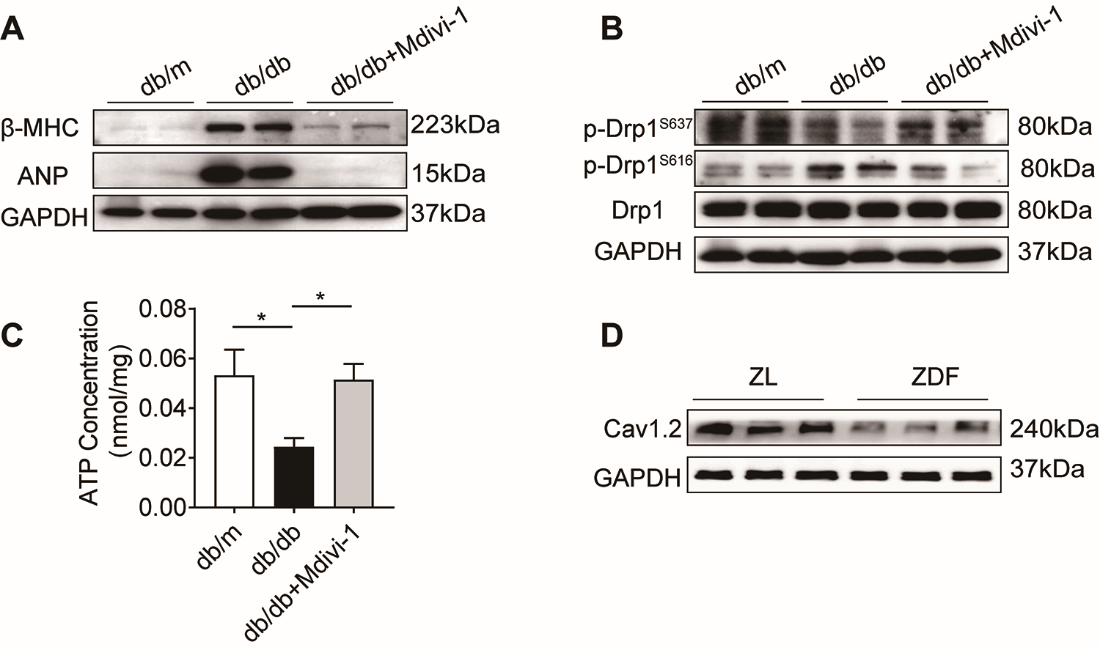
**

**Supplementary Fig S1.** **The effect of Mdivi-1 on mitochondrial morphology, ATP production and cardiac hypertrophy in diabetes mellitus.** (A, B) Western blotting images showing β-MHC, ANP, p-Drp1^S637^, and p-Drp1^S616^ protein levels in the ventricular tissue of diabetic mice injected with 10mg/kg Mdivi-1. (C) Changes in total ATP level in the ventricular tissue of diabetic mice injected with 10mg/kg Mdivi-1 (n = 3). (D) Western blotting images showing Cav1.2 protein level in the ventricular tissue of ZL and ZDF rats (n = 3). Data are shown as Mean ± SEM. ^＊^*P* < 0.05.
